# Future Inpatient Cost Burden of Laryngeal Cancer in Romania: Aging, Residence, and Prevention Scenarios in a Nationwide Ecological Study

**DOI:** 10.3390/healthcare14132007

**Published:** 2026-07-06

**Authors:** Andreea-Mihaela Banța, Livia Stanga, Ingrid-Denisa Barcan, Anda-Ioana Morgovan, Alexandru Orasan, Bogdan Hîrtie, Nicolae-Constantin Balica, Karina-Cristina Marin, Mihaela-Cristina Negru, Delia Ioana Horhat, Marius Papurica

**Affiliations:** 1 Department of ENT, “Victor Babes” University of Medicine and Pharmacy Timisoara, 300041 Timisoara, Romania; andreea.banta@umft.ro (A.-M.B.); anda.morgovan@umft.ro (A.-I.M.); alexandru.orasan@umft.ro (A.O.); bogdan.hirtie@umft.ro (B.H.); 2 Department of Otolaryngology, “Victor Babes” University of Medicine and Pharmacy Timisoara, 300041 Timisoara, Romania; balica@umft.ro (N.-C.B.); marin.karina@umft.ro (K.-C.M.); mihaela.negru@umft.ro (M.-C.N.); horhat.ioana@umft.ro (D.I.H.); 3 Methodological and Infectious Diseases Research Center, Department of Infectious Diseases, “Victor Babes” University of Medicine and Pharmacy Timisoara, 300041 Timisoara, Romania; stanga.livia@umft.ro; 4 Anaesthesia and Intensive Care Research Center, Faculty of Medicine, “Victor Babes” University of Medicine and Pharmacy, Eftimie Murgu Square 2, 300041 Timisoara, Romania; papurica.marius@umft.ro

**Keywords:** laryngeal neoplasms, economics, healthcare costs, hospitalization, statistics and numerical data, aged, Romania, epidemiology

## Abstract

**Background and Objectives:** Laryngeal cancer imposes substantial inpatient costs in middle-income health systems. We described recent trends in laryngeal cancer hospitalizations in Romania, estimated hospital expenditure, and modeled the impact of prevention scenarios to 2035. **Methods:** We conducted an ecological analysis of 20,056 discharges diagnosed as laryngeal cancer (ICD-10 C32.x) between 2019 and 2023. Each episode was assigned a mean unit cost of 1100 USD, with age- and residence-specific adjustments, and re-priced using EU (3900 USD) and US (30,000 USD) tariffs. We derived annual costs and projected 2035 expenditure under three prevention scenarios. **Results:** Annual discharges fell from 5408 in 2019 to 3151–3212 in 2020–2021, then rose to 4409 in 2023, while inpatient spending ranged from 3.47 to 5.95 million USD, reaching 4.85 million USD in 2023 (22,200 USD/100,000 population). Re-pricing 2023 activity at EU and US unit costs yielded counterfactual totals of 17.20 and 132.27 million USD. Older adults (≥65 years) generated 47.6% of discharges and 49.1% of spending in 2023, and urban residence increased the odds of age ≥65 by 48% (OR 1.48, 95% CI 1.40–1.57). Without new prevention, costs are projected to reach 12.60 million USD by 2035; a 30% smoking reduction and a combined package including radon mitigation and dysphonia screening would lower 2035 costs to 10.33 and 9.07 million USD. **Conclusions:** Demographic aging and sustained case volume will markedly increase hospital costs, while prevention scenarios are associated with lower projected inpatient expenditure.

## 1. Introduction

Laryngeal cancer is a relatively uncommon malignancy, but its impact is disproportionate because of its central role in phonation, airway protection, and social interaction. Loss of voice, chronic tracheostomy, and swallowing impairment can dramatically reduce quality of life and work capacity, even in patients who are technically “cured”. Globally, laryngeal cancer accounted for more than 209,000 new cases and 123,000 deaths in 2019, with 3.26 million disability-adjusted life years, and Central Europe shows some of the highest age-standardized incidence rates worldwide [1]. Recent analyses suggest that although age-standardized rates are slowly declining, the absolute number of cases will continue to rise to 2035 due to population growth and aging, with tobacco, alcohol, and occupational exposures remaining dominant drivers [1,2]. In this context, laryngeal cancer generates not only clinical but also considerable economic burden through repeated hospitalizations, complex surgery, radiochemotherapy, and prolonged rehabilitation.

In the United States, laryngeal cancer represents about 0.6% of all new cancers, with an estimated 13,020 new cases and 3910 deaths projected for 2025 [3,4]. Incidence rates have fallen over recent decades, largely reflecting reductions in smoking; however, mortality has not declined to the same extent, leading to a relative increase in case-fatality [3]. Contemporary SEER data indicate an overall incidence of 2.5 per 100,000 and a death rate of 0.9 per 100,000, with a median age at diagnosis of 66 years and a 5-year relative survival of around 62% [4]. These epidemiologic patterns translate into substantial health-system costs: head and neck cancer (HNC) patients typically require multimodal therapy, intensive acute care, and long-term surveillance, with higher rates of comorbidity and functional disability than many other solid tumors [5].

Economic evaluations from high-income settings underline the intensity and persistence of this financial burden. A systematic review of HNC costs estimated annual direct medical expenditures in the range of roughly USD 5000–15,000 per patient in US claims datasets, with higher costs in recurrent/metastatic disease and in surgically treated patients [5]. Medicare-based analyses show marked geographic variation in episode costs for advanced HNC, reflecting differences in treatment intensity and care pathways across US regions [6]. Survivors of HNC have higher median annual medical expenses and a greater share of their income spent on healthcare than survivors of other cancers, indicating a distinctive pattern of “financial toxicity” [7]. Even in universal-coverage systems, prospective studies document substantial out-of-pocket costs for travel, accommodation, medications, dental care, and ancillary services; for example, Canadian HNC patients undergoing chemoradiation incur median out-of-pocket expenditures of around CAD 1400–1600 over a single treatment course, with persistent costs and income loss during follow-up [8].

Across Europe, the macroeconomic footprint of cancer care is substantial and highly heterogeneous. The total economic burden of cancer in the EU-28 was estimated at EUR 199 billion in 2018, of which around 40% reflected direct healthcare costs, with head and neck sites among the more resource-intensive cancers because of high inpatient, surgical, and radiotherapy utilization [9,10,11]. Central and Eastern European countries tend to have higher incidence and mortality from tobacco-related cancers, including laryngeal cancer, but lower per capita healthcare spending and more constrained capacity for early diagnosis and supportive care [1,11]. The EU Country Cancer Profile for Romania highlights that per capita cancer care spending remains among the lowest in the EU, while avoidable mortality from preventable and treatable cancers is comparatively high, signaling underinvestment and inefficiencies along the care continuum [12]. These structural constraints amplify the economic consequences of each laryngeal cancer case for both patients and the health system.

Romania’s healthcare system is characterized by chronic underfunding, workforce shortages, and pronounced urban–rural disparities, all of which shape access to timely oncologic care [13,14]. Health expenditure as a share of GDP and per capita health spending lag behind EU averages, and hospital-centered care continues to dominate, with limited development of integrated rehabilitation and survivorship services [13,14]. Within this context, laryngeal cancer occupies a prominent position among male tobacco-related malignancies. A recent national analysis showed that Romania remains a high-burden country for laryngeal cancer mortality, with persistent excess in men and marked regional variation, despite modest declines in age-standardized mortality rates and projections suggesting only partial improvement by 2030 [15]. These patterns imply ongoing high demand for complex otolaryngology, oncology, and intensive-care services, with significant implications for hospital budgets and for patients’ household finances.

This study aimed to quantify the inpatient economic burden of laryngeal cancer hospitalizations in Romania using nationwide administrative discharge data, with attention to demographic aging and rural–urban residence. Specifically, we sought to: (i) describe national trends in laryngeal cancer hospitalizations and mortality (2019–2023); (ii) estimate annual inpatient expenditure under Romanian tariff assumptions and contextualize spending via EU/US counterfactual re-pricing; (iii) evaluate stratum-level differences in the age composition of discharges by residence and tumor subsite; and (iv) present transparent “what-if” projections of inpatient costs to 2035 under alternative prevention scenarios. The primary contribution is a transparent national enumeration and scenario-based budgeting framework in a setting where registry linkage and micro-costing are not routinely available.

To make the contribution explicit, the revised results are positioned as aggregate budget evidence rather than patient-level risk estimates: the manuscript links national discharge counts, an aging inpatient case mix, residence-related utilization differences, and conservative tariff assumptions to show how prevention scenarios may change future inpatient budget pressure. This clarification also explains why the study is clinically relevant despite using ecological administrative data.

### Literature Review and Research Gap

The available literature on laryngeal cancer burden can be grouped into three partially overlapping domains: epidemiology and preventable risk, clinical-resource intensity, and cost-of-illness evaluation. Epidemiologic studies consistently identify tobacco and alcohol exposure as dominant preventable determinants, while more recent global analyses emphasize that declining age-standardized rates may coexist with stable or rising absolute case numbers because the affected population is aging. This distinction is essential for health-system planning because hospital budgets respond to absolute activity volumes and case complexity rather than to age-standardized rates alone [1,2,3,4].

A second body of evidence concerns clinical-resource intensity. Laryngeal cancer often requires diagnostic endoscopy, imaging, surgery, airway procedures, tracheostomy care, radiotherapy or chemoradiotherapy, management of nutrition and swallowing impairment, and repeated post-treatment surveillance. Consequently, even when admission counts are lower than for more common cancers, each episode may involve complex multidisciplinary care. International cost studies show that surgical treatment, recurrent or metastatic disease, multimorbidity, and older age increase expenditure, but most of this literature comes from high-income settings with more detailed billing or registry linkage than is routinely available in Romania [5,6,7,8,9,10,16,17,18,19,20,21,22,23].

The third relevant domain is geographic inequality. Rurality, lower socioeconomic status, and distance from tertiary oncology or otolaryngology services have been linked to delayed diagnosis, different treatment access, and survival disparities in head-and-neck cancers. However, administrative discharge data capture treated hospital episodes rather than the full underlying incidence of disease. Therefore, residence patterns in discharge datasets should be interpreted as health-system utilization patterns, not as direct evidence that the true biological incidence is higher or lower in rural or urban populations [24,25,26].

For Central and Eastern Europe, the literature remains more limited. Romania combines a high tobacco-related cancer burden, relatively low per capita health expenditure, and a hospital-centered delivery model. Prior Romanian work has described mortality trends and regional variation in laryngeal cancer, but less is known about how inpatient utilization translates into provider-side hospital costs or how aging and residence structure these costs at the national level [12,13,14,15].

The present study addresses this gap by using a transparent ecological framework based on nationwide discharge strata. The novelty is not an individual-level prediction model but a national budget-enumeration approach that combines observed discharge volumes, age/residence structure, conservative provider-side unit-cost assumptions, international re-pricing benchmarks, and deterministic prevention scenarios. This approach is appropriate when micro-costing and patient-level linkage are unavailable, provided that all interpretations remain explicitly aggregate and scenario-based.

Taken together, previous studies support the need for a national inpatient-cost perspective that is transparent about its limits. Epidemiologic burden alone does not indicate how many hospital episodes must be financed, clinical cost studies from high-income systems may not transfer directly to Romanian tariffs, and rural–urban comparisons can be distorted when untreated or delayed cases are not captured in discharge records. The present analysis therefore uses administrative data for what they can robustly provide: national utilization, aggregate age and residence structure, coding patterns, and internally consistent budget scenarios.

## 2. Materials and Methods

### 2.1. Study Design and Setting

We conducted a nationwide, retrospective ecological study using routinely collected administrative data on hospital discharges and mortality due to laryngeal cancer in Romania between 1 January 2019 and 31 December 2023. The study period was chosen to align hospital-discharge records with mortality and population datasets compiled using consistent coding practices and to capture the pre-pandemic and post-pandemic phases of health-system activity. The analysis followed STROBE recommendations for observational studies, with particular attention to transparent reporting of data sources, transformations, and statistical methods.

The study population comprised all inpatient episodes with a principal diagnosis of malignant neoplasm of the larynx, identified through International Classification of Diseases, Tenth Revision (ICD-10) codes C32.0 through C32.9. Discharges originated from public and private hospitals reporting to the national information system, including tertiary referral centers, regional hospitals, and smaller county facilities.

Analytic steps were structured to address the study objectives: descriptive trends quantify national utilization; stratified summaries describe demographic and residence patterns; grouped regression evaluates stratum-level age composition differences; cost enumeration translates utilization into inpatient spending; and scenario analyses illustrate potential 2035 budget impacts under alternative prevention assumptions.

In this study, “ecological pattern” refers to variation observed across aggregated discharge strata, including calendar year, residence, age category, sex, hospital/county reporting units, and ICD-10 laryngeal subsite groups. The unit of inference is therefore the stratum or national annual total rather than the individual patient. This design allows national utilization and budget patterns to be described when individual identifiers are unavailable, but it does not support individual-level causal inference.

### 2.2. Data Sources and Variable Definitions

Hospital-discharge data were extracted from a national dataset that records, for each combination of year, county, hospital, diagnosis code, sex, rural/urban residence, and age group, the number of continuous-hospitalization discharges. Age was recorded in predefined bands: under 1 year, 15–19, 20–24, 30–34, 35–39, 40–44, 45–49, 50–54, 55–59, 60–64, 65–69, 70–74, 75–79, 80–84, and ≥85 years. For analytic simplicity, these bands were collapsed into three categories representing early adulthood and mid-life (<55 years), pre-retirement (55–64 years), and older age (≥65 years). For each stratum, the counts in the detailed age bands were summed to check consistency with the reported total number of discharges. The ≥65 cut-point was selected a priori because it is a standard threshold for older-age policy planning and because older adults account for the largest share of inpatient spending in our 2023 decomposition.

Indicator selection was guided by three theoretical considerations. First, annual discharge volume is a direct measure of inpatient service utilization and is therefore the principal driver of provider-side budget impact. Second, age structure is clinically relevant because older patients more often have multimorbidity, frailty, longer recovery, and higher perioperative and supportive-care needs. Third, residence was included as a health-services access indicator because rural and urban populations may differ in proximity to specialist otolaryngology, oncology, imaging, rehabilitation, and follow-up services. Tumor subsite was retained because clinical pathways and coding specificity may differ by localization, but subsite findings were treated as descriptive because C32.9 coding was frequent.

Tumor subsite was derived from the three-digit ICD-10 extension: C32.0 (glottic), C32.1 (supraglottic), C32.2 (subglottic), C32.3 (laryngeal cartilage), C32.8 (overlapping lesion of larynx), and C32.9 (larynx, unspecified). These were grouped into five clinically meaningful categories: glottic (C32.0), supraglottic (C32.1), subglottic (C32.2), transglottic/other specified (C32.3 and C32.8), and unspecified (C32.9). Sex was coded as male or female, and residence was coded as rural or urban based on the official classification of the patient’s domicile. The primary outcome at the discharge-stratum level was the proportion of patients aged ≥65 years, constructed as the ratio between the number of discharges in the 65–69, 70–74, 75–79, 80–84, and ≥85 age bands and the total number of discharges in that stratum.

Age-standardized mortality rates for malignant laryngeal tumors were obtained from the national mortality compilation, which provides, for each calendar year, the directly standardized mortality rate for Romania as a whole, calculated per 100,000 population. These rates were used to build an annual series for 2019–2023. The dataset does not include patient identifiers; therefore, analyses are conducted at the discharge-episode level, and repeated admissions by the same individual cannot be identified.

### 2.3. Economic Evaluation and Cost Modeling

The economic component of this study was conducted from the hospital provider perspective, restricted to direct medical costs associated with each inpatient episode for laryngeal cancer. Because individual-level billing data were not available, we used a top–down modeling approach in which each discharge was assigned a plausible mean cost per episode, and total national expenditure was derived by multiplying this unit cost by the observed number of discharges.

For reproducibility, costs were computed with a simple deterministic identity: total inpatient cost equals the number of discharges multiplied by the assigned mean cost per discharge. For age- and residence-specific decompositions, the same identity was applied within each stratum and then summed to the national total. Counterfactual EU and US costs were obtained by applying the benchmark unit prices to the observed Romanian volumes only; no assumptions were made about international differences in clinical pathways, length of stay, or case mix.

The exact calculation was Cost(y,s) = Discharges(y,s) × Unit cost(s), where y denotes calendar year and s denotes the relevant stratum when age, residence, or scenario decompositions were performed. National totals were obtained by summing Cost(y,s) across strata. Scenario totals used the same identity after applying the stated discharge multipliers; therefore, every value in the tables can be reproduced from the reported discharge counts and unit-cost assumptions.

All costs were expressed in US dollars (USD) and standardized to a single price year (2023) using official exchange rates and assuming stable tariffs over the 2019–2023 period. Initial costs were estimated for the lower end of the price range and for the respective year 2019. Anchoring of the Romanian unit cost: In Romania, inpatient hospitalizations are commonly financed through a DRG-type case-payment approach in which reimbursement for a “resolved case” is derived from the hospital-specific tariff per weighted case (TCP) and the hospital case mix index (ICM), yielding a contract-based value per case (Tarif pe caz rezolvat). Publicly available contract documents from Romanian hospitals report TCP/ICM values that translate into resolved-case payments on the order of several thousand RON for high-complexity services; as an illustration, one 2023 contracted example reports ICM 2.8043 and TCP 2086 RON, corresponding to a resolved-case tariff of 5849.77 RON. Another publicly available contract document reports a hospital TCP of 2465 RON for the second semester of 2024. Given (i) these contract-based magnitudes, (ii) the likelihood that laryngeal cancer admissions at ENT/oncology providers involve above-average DRG weights and perioperative resource use, and (iii) the absence of case-level DRG weights, length-of-stay, and procedure codes in the aggregated dataset, we selected 1100 USD as a conservative, rounded base-case provider-side episode cost and quantified uncertainty via sensitivity analyses.

The 1100 USD base-case should therefore be read as a conservative national accounting assumption rather than as a patient-level bill or a full societal cost. It is intended to approximate average provider-side inpatient reimbursement in the absence of case-level DRG weights, procedures, length of stay, intensive-care days, radiotherapy exposure, readmissions, or outpatient costs. This choice makes the Romanian estimates transparent and reproducible while deliberately avoiding over-precision.

Exchange-rate anchoring of Romanian tariff examples. To improve the interpretability of the cited contract magnitudes, we express the illustrative resolved-case tariff in USD using a realistic 2023–2024 exchange-rate range (≈4.5–4.6 RON/USD). A resolved-case value of 5849.77 RON therefore corresponds to approximately 1272–1300 USD (5849.77/4.6 to 5849.77/4.5). This conversion indicates that the contracted resolved-case payments reported in publicly available hospital documents are of the same order of magnitude as our 1100 USD base-case. Importantly, laryngeal oncology admissions may carry higher DRG weights than average resolved cases; however, the administrative dataset is aggregated and lacks DRG weight, procedure, and length-of-stay fields. We therefore treat 1100 USD as a conservative provider-side episode cost for national budget enumeration and emphasize uncertainty through one-way sensitivity analyses (±20% and ±50%) rather than presenting it as a micro-costed estimate. Because laryngeal-oncology admissions that include laryngectomy, tracheostomy, radiotherapy-related care, or higher DRG relative weights may exceed the illustrative resolved-case tariff, the retained 1100 USD value should be interpreted as a deliberately conservative mean provider-side episode cost for aggregated national budgeting rather than as a micro-cost for a specific treatment pathway.

For international comparison, we applied two benchmark unit costs without altering Romanian activity volumes: (i) an EU reference cost of 3900 USD per laryngeal/head-and-neck episode, and (ii) a US reference cost of 30,000 USD per initial laryngeal cancer hospitalization. EU and US unit-cost benchmarks were used only for counterfactual re-pricing of Romanian discharge volumes to contextualize the magnitude of resource use under alternative tariff environments; they are not interpreted as directly comparable “true costs” because underlying case mix, costing methods (micro-costing vs. top–down), and payment structures differ across health systems. Accordingly, we treat these benchmarks as contextual comparators rather than as calibration targets [16,17,18,19,20].

We then constructed several derived economic indicators: (a) total annual inpatient cost in Romania (2019–2023), (b) cost per 100,000 population, (c) age-stratified and rural–urban cost breakdowns, and (d) counterfactual expenditures had the same Romanian discharges been reimbursed at EU or US unit costs. Uncertainty around these cost estimates was explored in scenario analyses, in which discharge volumes for 2035 were varied under three assumptions: no additional prevention (baseline scenario), a 30% reduction in adult smoking prevalence, and a combined package of smoking reduction plus radon mitigation and systematic dysphonia screening. For each scenario, we calculated the projected total inpatient cost in Romania and the incremental cost difference relative to the observed 2023 baseline. All cost modeling was implemented using deterministic spreadsheets; no probabilistic sensitivity analysis was performed, so the results should be interpreted as illustrative ranges rather than precise budget-impact estimates.

Because C32.9 (“unspecified”) accounted for approximately half of discharges, subsite-specific comparisons should be interpreted cautiously as they reflect documentation and coding practices rather than true clinical composition. Therefore, we treated subsite analyses as descriptive and performed sensitivity checks, excluding C32.9 and/or omitting subsite as a predictor.

### 2.4. Deterministic Scenario Model (2023–2035)

Projections were generated as deterministic scenario analyses intended to illustrate plausible ranges of inpatient budget impact rather than to provide epidemiologic forecasts. The model starts from observed 2023 activity (discharges and base-case unit cost) and projects annual discharges to 2035 under a baseline “no new policies” trajectory and two prevention scenarios. To reflect population aging, we referenced European Commission aging projections for Romania (including projected increases in old-age dependency metrics over the coming decades). Because the administrative dataset is aggregated and lacks stage, treatment intensity, and patient identifiers, we did not implement a full incidence model; instead, we applied transparent scenario multipliers to discharge volumes and report uncertainty in sensitivity analyses. Demographic aging context was referenced using the European Commission Ageing Report (Eurostat-based projections). Because parameter distributions were not available (no micro-costing or patient-level resource-use data), probabilistic sensitivity analysis was not performed; instead, we conducted deterministic one-way and multi-way sensitivity analyses varying key parameters across plausible ranges.

The baseline trajectory is intentionally conservative in interpretation: it extrapolates a short, disruption-prone 2019–2023 period that includes the COVID-19 contraction and subsequent rebound in hospital activity. We therefore do not present the 2035 values as a forecast of true incidence. Instead, they represent internally consistent budget scenarios that test how inpatient expenditure would change if recent utilization momentum and specified prevention effects were carried forward.

Screening-related stage shift could plausibly increase some treatment expenditures even if mortality improves; because stage and treatment data are unavailable, this mechanism is not modeled and projections represent simplified “what-if” inpatient budget scenarios.

Because procedure-level costing is unavailable, we did not assign empirical subsite-specific unit costs. We instead tested an illustrative sensitivity scenario applying plausible multipliers by subsite (glottic lower, supraglottic/transglottic higher) to assess robustness.

We varied the Romanian unit cost by ±20% and ±50% to reflect uncertainty in average inpatient resource use across episode types. We also varied the assumed prevention impact by testing alternative discharge-reduction ranges by 2035 (20–40% relative reductions) to illustrate dependence on intervention effectiveness, uptake, and lag assumptions. Results are summarized in Table 1.

Projection equations and scenario multipliers: Baseline projected discharges were calculated as D(t, baseline) = 4409 × (1 + g)^(t − 2023), where 4409 is the observed number of discharges in 2023 and g is the constant annual growth rate required to reach 11,454 discharges in 2035. Solving this identity yields an implied compounded annual growth rate of approximately 8.3%. Year-by-year projected baseline discharges for 2024–2035 are reported in Table A1.

Prevention scenarios were implemented as deterministic multiplicative reductions applied to the baseline trajectory. For each future year, D(t, scenario) = D(t, baseline) × m(t), where the multiplier m(t) declines gradually over time to reflect implementation and lag rather than an instantaneous effect. The smoking-reduction scenario uses a multiplier that falls from 0.985 in 2024 to 0.820 in 2035, yielding 9392 projected discharges in 2035 (an 18% reduction versus baseline). The combined package uses a multiplier that falls from 0.977 in 2024 to 0.720 in 2035, yielding 8247 projected discharges in 2035 (a 28% reduction versus baseline).

### 2.5. Statistical Analysis

Descriptive statistics summarized the number of laryngeal cancer discharges by year, sex, residence, age category, and tumor subsite. Continuous variables (such as age-standardized mortality rates) were expressed as means and standard deviations, while categorical variables were expressed as counts and percentages. Differences in the distribution of sex and residence across calendar years were evaluated using chi-square tests of independence applied to contingency tables with discharge counts as cell entries. For age structure, we compared the three age categories across rural and urban areas using chi-square tests and reported *p*-values for global differences.

To investigate determinants of older age at discharge, we fitted a binomial logistic regression model with grouped data. For each hospital demographic stratum, the number of “events” was defined as the count of discharges aged ≥65 years and the number of “non-events” as the count of discharges aged <65 years. The dependent variable was the proportion of age ≥65, modeled with a logit link. Independent variables included calendar year (coded as a continuous variable with 2019 as the reference), sex, residence, and tumor subsite. The model was fitted using generalized linear modeling with a binomial family and frequency weights equal to the total number of discharges in each stratum. Odds ratios (ORs) with 95% confidence intervals (CIs) were derived by exponentiating model coefficients. All regression results reflect stratum-level composition and do not support individual-level causal inference (risk of ecological fallacy).

Model adequacy was assessed using grouped binomial goodness-of-fit metrics and sensitivity to alternative specifications. We examined the ecological relationship between hospital-discharge volume and national age-standardized mortality by constructing a five-point annual series (2019–2023) of total discharges and mortality rates. Pearson correlation assessed linear association, while Spearman rank correlation assessed monotonic association. Given the small number of time points, these correlations were interpreted cautiously as exploratory. A two-sided *p*-value below 0.05 was considered statistically significant throughout. All analyses were conducted using reproducible code in a statistical environment capable of handling grouped binomial and contingency-table analyses. The grouped binomial model estimates differences in the proportion of discharges aged ≥65 across strata and should not be interpreted as an individual-level risk model. To assess potential clustering of discharge strata within hospitals/counties, we performed robustness checks using cluster-robust standard errors and repeated analyses excluding sparse strata.

Robustness to unspecified subsite coding (C32.9): Given the high prevalence of C32.9 (“unspecified”), we conducted sensitivity analyses to assess whether model inferences were driven by coding composition. We refit the grouped binomial logistic regression (i) excluding C32.9 strata, (ii) excluding tumor subsite as a covariate, and (iii) restricting it to specified subsites only. Across these specifications, the urban–rural difference and the calendar-year aging trend remained directionally consistent. The corresponding results are summarized in Table A2, which shows that the urban-residence effect and the calendar-year aging trend remained positive and statistically significant across the alternative specifications requested by the reviewer.

For the primary grouped binomial model, we report standard goodness-of-fit summaries: residual deviance on degrees of freedom; Pearson χ^2^\chi^^2^χ^2^ on degrees of freedom, corresponding to an estimated dispersion. These statistics are reported descriptively to document model fit for grouped data and do not alter the ecological interpretation.

For the primary grouped-binomial model, goodness-of-fit was evaluated descriptively using residual deviance and Pearson chi-square relative to degrees of freedom, together with the specification checks reported above. Because the model was intended to characterize stratum-level age composition rather than to generate individual-level predictions, these diagnostics were interpreted as supportive checks; in this context, deviance and Pearson statistics close to their corresponding degrees of freedom were considered reassuring rather than decisive.

## 3. Results

Although men represent the vast majority of hospitalizations throughout, the sex distribution varies significantly across years (χ^2^ = 13.68, *p* = 0.008), driven by a small but measurable fluctuation in the female share during the pandemic and recovery period (Table 2).

The rural–urban split changes modestly but significantly over time (χ^2^ = 9.65, *p* = 0.047), indicating that the pandemic-era contraction and subsequent rebound were accompanied by a small shift toward a higher rural proportion (Table 3).

Urban residence shows a clearly older case mix (higher proportion aged ≥65 and lower proportion <55) compared with rural residence, aligning with the regression finding that urban strata have higher odds of being in the ≥65 category (Table 4).

Subsite composition changes strongly across years (χ^2^ ≈ 133.5, *p* < 0.001), consistent with meaningful temporal variation in coding/subsite mix—most notably the persistently large “unspecified” fraction and shifts in the transglottic/other and unspecified categories across the period (Table 5).

Using 2019 as the reference year, and glottic tumors, female sex, and rural residence as baseline categories, several patterns emerged (Table 6). Calendar year showed a robust positive association: each one-year increase was associated with a 7% relative increase in the odds of age ≥65 (OR 1.07, 95% CI 1.06–1.09, *p* < 0.001), indicating progressive aging of the inpatient laryngeal cancer population over the short five-year window. Residence was also an independent determinant: urban discharges had 1.48 times higher odds of involving patients aged ≥65 years than rural discharges (95% CI 1.40–1.57, *p* < 0.001), even after controlling for sex and subsite, corroborating the descriptive findings. By contrast, sex did not significantly influence age structure: male discharges had only slightly higher odds of age ≥65 than female discharges (OR 1.06, 95% CI 0.94–1.19, *p* = 0.35). Compared with glottic tumors, supraglottic lesions were associated with lower odds of age ≥65 (OR 0.59, 95% CI 0.50–0.70, *p* < 0.001), and transglottic/other specified lesions also showed reduced odds (OR 0.79, 95% CI 0.72–0.87, *p* < 0.001). Unspecified tumors had modestly lower odds (OR 0.90, 95% CI 0.83–0.98, *p* = 0.021). Strata corresponding to urban residence showed a higher odds of a discharge being in the ≥65 category, reflecting differences in the age composition of aggregated discharge strata. This pattern remained materially unchanged in the sensitivity analyses excluding unspecified C32.9 strata, omitting tumor subsite, or restricting the model to specified subsites only (Table A2).

Discharges declined sharply from 5408 in 2019 to 3151 in 2020 and 3212 in 2021, then climbed to 3876 in 2022 and 4409 in 2023. In contrast, the age-standardized mortality rate showed more modest variation, from 4.19 per 100,000 in 2019 to 4.21 in 2020, then decreasing to 3.76 in 2021 and 3.70 in 2022, before rising slightly to 3.98 in 2023. Pearson’s correlation coefficient between discharge counts and mortality over these five years was 0.35 (*p* = 0.56), indicating a weak, non-significant positive linear association. Spearman’s rank correlation coefficient was −0.10 (*p* = 0.87), suggesting no consistent monotonic relationship (Table 7).

Table 8 summarizes the national inpatient costs of laryngeal cancer hospitalizations in Romania between 2019 and 2023 under three pricing assumptions. Using the Romanian base tariff of 1100 USD per episode, total annual expenditure fell from 5.95 million USD in 2019 to 3.47–3.53 million USD in 2020–2021, then rose again to 4.26 million USD in 2022 and 4.85 million USD in 2023, mirroring the pandemic-related collapse and subsequent recovery of discharge volumes. When expressed per 100,000 population, Romanian inpatient costs declined from 26,800 USD in 2019 to 15,600–16,000 USD in 2020–2021, then increased to 19,400 USD in 2022 and 22,200 USD in 2023. Applying an EU benchmark price of 3900 USD per episode yields counterfactual costs of 21.09–17.20 million USD per year, while a US benchmark of 30,000 USD per initial hospitalization generates much higher hypothetical expenditures of 162.24–132.27 million USD. Across all years, EU-level unit costs would be roughly 3.5 times Romanian spending, and US prices about 27 times higher for the same activity volumes.

Table 9 decomposes the 2023 Romanian inpatient cost of laryngeal cancer by age group using age-specific unit costs. Patients younger than 55 years generated 733 discharges priced at 1000 USD per episode, for a total Romanian expenditure of 733,000 USD and counterfactual EU and US costs of 2.86 million and 21.99 million USD, respectively. The 55–64 year group accounted for 1579 discharges with a mean Romanian cost of 1100 USD, resulting in 1.74 million USD of national spending and hypothetical EU and US totals of 6.16 million and 47.37 million USD. Patients aged ≥65 years generated the largest financial burden: 2097 discharges with a higher Romanian unit cost of 1135 USD produced 2.38 million USD in domestic expenditure and counterfactual EU and US costs of 8.18 million and 62.91 million USD.

The age-specific totals in Table 9 are derived directly from the observed 2023 discharge counts multiplied by the pre-specified unit-cost assumptions for each age band. These adjustments are deterministic accounting weights used for budget decomposition; they are not estimates from individual billing records.

Table 10 contrasts rural and urban inpatient costs for laryngeal cancer in 2023. Rural residents accounted for 2305 discharges with a lower mean Romanian unit cost of 1050 USD, yielding a total expenditure of approximately 2.42 million USD. Urban residents generated slightly fewer discharges (2104) but had a higher mean unit cost of 1154.78 USD, resulting in a very similar total Romanian cost of about 2.43 million USD. Despite these different unit prices, the aggregate national inpatient cost (4.85 million USD) is almost evenly split between rural and urban areas. Under EU benchmark pricing, rural and urban discharges would translate into counterfactual totals of 8.99 million and 8.21 million USD, respectively, while US benchmark pricing would imply 69.15 million USD for rural and 63.12 million USD for urban cases.

The residence-specific totals in Table 10 were calculated analogously by multiplying rural and urban discharge counts by the corresponding deterministic residence-specific unit costs and then summing to the national 2023 total. These values describe aggregate provider-side budget allocation by domicile category, not causal cost differences between individual rural and urban patients.

Table 11 presents projected inpatient costs for laryngeal cancer discharges in 2035 under alternative prevention scenarios, compared with the observed 2023 baseline. The baseline row reproduces the 2023 situation: 4409 discharges, a mean Romanian cost of 1100 USD, and total inpatient expenditure of 4.85 million USD. If current trends continue without new policies, discharges are projected to rise to 11,454 in 2035, with Romanian inpatient costs increasing to 12.60 million USD—an incremental 7.75 million USD over 2023—and counterfactual EU and US costs of 44.67 million and 343.62 million USD. A 30% reduction in adult smoking would lower projected discharges to 9392 and reduce Romanian spending to 10.33 million USD (5.48 million USD above 2023), while a combined package of smoking reduction, radon mitigation, and dysphonia screening would further decrease discharges to 8247 and national costs to 9.07 million USD (4.22 million USD above 2023).

The steep 8.3% compound annual growth rate should not be read as a biological growth rate in laryngeal cancer incidence. It is the arithmetic rate needed to connect the observed 2023 utilization baseline with the selected 2035 no-policy scenario after the pandemic-period contraction and rebound. Thus, the scenario is best interpreted as a budget stress test showing what inpatient expenditure would look like if recent hospital utilization momentum persisted, not as a prediction that the true number of patients with laryngeal cancer will grow at that rate.

**Figure 1:**
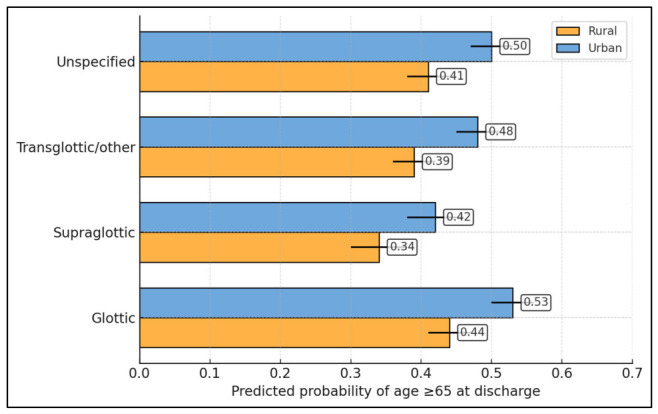
Predicted probability that a discharge belongs to the ≥65 age category by residence and tumor subsite (stratum-level model). This figure visualizes the age-composition differences that underpin the concentration of inpatient costs in older adults and the residence gradient in case mix.

Figure 1 displays modeled probabilities of being aged ≥65 years at discharge in 2023 by tumor subsite and residence, based on the multivariable logistic regression. For glottic cancers, the predicted probability of age ≥65 rises from 0.44 (95% CI 0.41–0.47) in rural residents to 0.53 (95% CI 0.50–0.56) in urban residents, illustrating the strong independent effect of urban residence. Supraglottic tumors show the lowest predicted age, with probabilities of 0.34 (95% CI 0.30–0.38) in rural and 0.42 (95% CI 0.38–0.46) in urban areas, consistent with their association with relatively younger patients. Transglottic/other specified lesions occupy an intermediate position (0.39 rural, 0.48 urban), while unspecified tumors approach glottic values (0.41 rural, 0.50 urban). Across all subsites, urban residence shifts the predicted probability of age ≥65 upward by 7–9 percentage points, and glottic or unspecified localization is consistently associated with an older case mix than supraglottic disease.

**Figure 2:**
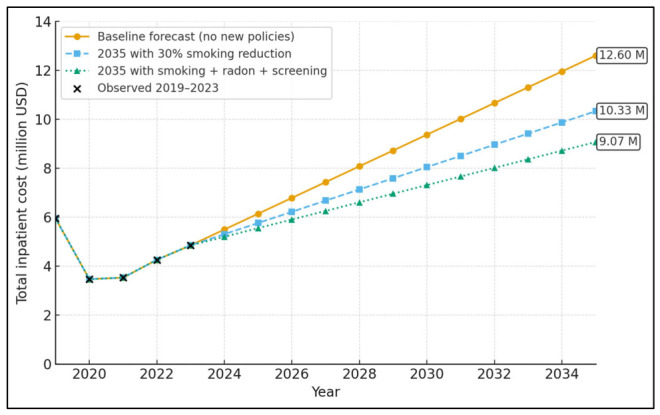
Observed and projected national inpatient costs of laryngeal cancer hospitalizations in Romania, 2019–2035, under three policy scenarios.

Figure 2 traces the evolution of national inpatient costs for laryngeal cancer discharges in Romania from 2019 to 2035 under three scenarios. The dark blue curve shows the observed cost compression during the pandemic (from 5.95 million USD in 2019 to 3.47–3.53 million USD in 2020–2021), followed by recovery to 4.26 million USD in 2022 and 4.85 million USD in 2023. Extrapolating current trends without additional prevention (baseline scenario) yields a steep ascent to approximately 12.60 million USD by 2035—more than a 2.5-fold increase over 2023. Introducing a 30% reduction in adult smoking prevalence flattens the orange dashed curve, lowering the 2035 cost to roughly 10.33 million USD and reducing the budget impact by 2.27 million USD compared with the baseline scenario. The green dotted line, representing combined smoking reduction, radon mitigation, and dysphonia screening, bends even more gently, with a projected 2035 cost of 9.07 million USD, corresponding to 3.53 million USD in savings relative to the no-policy baseline. The widening separation between lines after 2028 visually underscores how early prevention investments accumulate into substantial long-term cost avoidance, even though all scenarios share the same starting point in 2019–2023.

**Figure 3:**
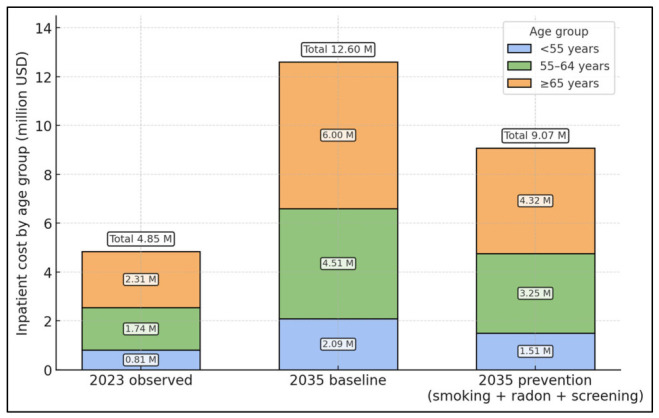
Age group decomposition of inpatient costs under three scenarios.

Figure 3 decomposes Romanian inpatient laryngeal cancer costs by age group for the 2023 observed baseline and two 2035 scenarios. In 2023, total inpatient spending was about 4.85 million USD, of which roughly 0.81 million USD (16.6%) occurred in patients younger than 55 years, 1.74 million USD (35.8%) in those aged 55–64 years, and 2.31 million USD (47.6%) in patients aged ≥65 years. Under the 2035 baseline scenario without new prevention, total costs rise to approximately 12.60 million USD, with contributions of 2.09 million, 4.51 million, and 6.00 million USD from the <55, 55–64, and ≥65 groups, respectively. In the 2035 combined prevention scenario, total expenditure falls to about 9.07 million USD, but the ≥65 group still accounts for 4.32 million USD, compared with 3.25 million USD for ages 55–64 and 1.51 million USD for <55. Across all scenarios, patients aged ≥65 years consistently consume around half of total inpatient spending, visually demonstrating that demographic aging is the dominant driver of future cost growth—even when effective smoking reduction, radon mitigation, and dysphonia screening flatten the overall discharge curve (as described in Table 12, Table 13, and Table 14).

To improve readability, the detailed sensitivity tables are interpreted as follows. The discharge-to-patient analysis shows that repeated admissions would increase the implied per-patient inpatient cost but would not alter the national provider-side budget total. The cluster-robust regression check widens confidence intervals but preserves the direction of the urban-residence and calendar-year aging associations. The EU/US range-based re-pricing demonstrates that the policy message does not depend on a single benchmark tariff because international counterfactual totals remain several-fold higher than the Romanian base-case under all tested values.

## 4. Discussion

### 4.1. Analysis of Findings

The Romanian cost estimates derived in this study are best interpreted in light of micro-costing data for laryngeal and head-and-neck surgery from other health systems. Itemized analyses of total laryngectomies in the United States report hospital costs in the tens of thousands of US dollars per admission, with operating room time, intensive care, and extended length of stay as major drivers [20]. Similar cost structures are seen in middle-income settings: in an Indian tertiary center, Chauhan et al. found that head-and-neck cancer treatment consumed a large share of annual household income, with radiotherapy, chemotherapy, and surgery together accounting for several thousand US dollars per patient even under public tariffs [21]. In Brazil, Milani et al. documented first-year direct healthcare costs for lip, oral cavity, and oropharyngeal cancers that again reach multiple thousands of dollars per case in a universal coverage system [22]. A recent Brazilian cost–consequence analysis that focused specifically on laryngeal cancer showed that different surgical and non-surgical strategies generate substantial but comparable inpatient and outpatient expenditures per episode [23]. Against this backdrop, the Romanian base tariff of 1100 USD per discharge appears conservative, reinforcing the interpretation of our EU and US “re-pricing” scenarios as lower-bound approximations of the true resource demand associated with laryngeal cancer hospitalizations rather than exaggerated upper limits.

Our finding that nearly half of the inpatient bills are generated by patients aged ≥65 years is consistent with international evidence that head-and-neck cancer care becomes disproportionately costly in older, multimorbid groups. The US cost-identification analysis of total laryngectomy found that post-operative complications, prolonged hospitalization, and rehabilitation needs substantially increased costs, factors that are more common in older patients with cardiovascular and pulmonary comorbidities [20]. Indian and Brazilian cost studies similarly show that advanced stage at diagnosis, the need for combined-modality treatment, and higher burden of treatment-related toxicity—all more frequent in older, heavily exposed smokers—are associated with steep cost increments per patient [21,22]. In this context, the Romanian pattern of progressively aging inpatients (OR 1.07 per calendar year for age ≥65) suggests that even if tariffs remain low, aggregate expenditure will rise as the case mix shifts toward those at highest risk of complications and re-admission. This reinforces the argument that geriatric-adapted perioperative pathways and early rehabilitation are not merely quality initiatives but central cost-containment strategies in an aging laryngeal cancer population.

The near-equal rural and urban contribution to discharge volume, combined with a markedly older urban case mix, offers an interesting contrast to emerging work on geographic and socioeconomic gradients in head-and-neck cancer. A large Finnish registry analysis showed persistent rural–urban and educational gradients in head-and-neck cancer incidence from 1977 to 2021, with higher incidence in low-education and rural populations [24]. A recent scoping review concluded that rurality is typically associated with a more advanced stage at diagnosis, longer travel distances, and fragmented survivorship care in head-and-neck cancer, although patterns vary by country and health-system organization [25]. At the same time, population-based US data indicate that lower neighborhood socioeconomic status is associated with worse survival in oral cavity and laryngeal cancers, independent of race and ethnicity [26]. Romania’s pattern is that of slightly higher discharge counts from rural areas but an older, potentially more comorbid urban inpatient case mix, reflecting a combination of rural under-diagnosis or delayed presentation, selective referral of complex cases to urban tertiary centers, and urban–rural differences in smoking or occupational exposure histories.

Clinically, the older urban inpatient case mix should not be interpreted as evidence that rural populations carry less disease. A plausible alternative explanation is under-diagnosis, delayed referral, or later presentation among rural residents, especially when early dysphonia, swallowing symptoms, or airway complaints do not rapidly reach specialist evaluation. Rural patients may also be hospitalized closer to the point of advanced symptom burden, while urban residents may have more frequent surveillance admissions and better access to tertiary otolaryngology services. This interpretation is consistent with the ecological nature of the dataset and supports prevention strategies that improve early symptom recognition and referral pathways outside of large urban centers.

Our prevention scenarios, which assume a 30% reduction in adult smoking and additional gains from radon mitigation and dysphonia screening, are supported by recent epidemiologic and health-economic evidence. A meta-analysis of 65 studies found that smoking cessation roughly halves the risk of head-and-neck cancer, with substantial risk reductions for laryngeal tumors after sustained abstinence [27]. Systematic review of trials in head-and-neck cancer patients suggests that multi-component smoking-cessation programs combining pharmacotherapy and intensive counseling improve quit rates compared with usual care, although high-quality evidence remains limited [28]. From an economic perspective, modeling studies in mixed cancer populations show that embedding structured cessation programs into oncology care is highly cost-effective, with incremental cost-effectiveness ratios well below commonly accepted thresholds [29,30]. These data make it plausible that a sustained, system-wide reduction in smoking prevalence of the magnitude modeled here would not only moderate future laryngeal cancer incidence but also yield net savings once avoided treatment costs and improved outcomes are accounted for. The Romanian estimates therefore align with a growing consensus that tobacco control in cancer patients and high-risk groups is among the most economically attractive interventions available to oncology services.

From a medical perspective, prevention is relevant to hospital costs because it may reduce not only the number of incidental smoking-related tumors but also the frequency of airway emergencies, advanced-stage resections, tracheostomy-related care, malnutrition, aspiration, and prolonged rehabilitation. Similarly, systematic evaluation of persistent dysphonia can shift some patients toward earlier diagnostic work-up, although the present model cannot quantify stage shift directly because stage and treatment modality were not available in the administrative dataset.

Finally, our ecological modeling adds a Central and Eastern European perspective to a literature that has been dominated by Western European and North American cost-of-illness studies. While registry-based analyses from Finland, scoping reviews on rurality, and US neighborhood SES studies highlight how structural disadvantage and geography shape head-and-neck cancer risk and outcomes [24,25,26], few studies have integrated these dimensions into forward-looking economic projections. Romania’s combination of high laryngeal cancer mortality, a rapidly aging population, and low per capita health spending means that each prevented case yields a larger relative gain in both survival and fiscal space than in wealthier EU countries. At the same time, our findings underscore the limits of hospital-centric policies: without upstream investment in tobacco control, occupational protection, radon remediation, and primary-care-based voice and airway assessment, demographic pressures will eventually overwhelm even efficiency gains in inpatient care. By quantifying how much of the future hospital bill is driven by aging versus modifiable risk factors, this study provides a framework that can be adapted to other tobacco-related cancers and to neighboring health systems facing similar demographic and budgetary constraints.

Although this analysis focuses on inpatient provider-side costs, head-and-neck cancer is frequently associated with financial toxicity through mechanisms that are largely outside the scope of our discharge-based costing, such as out-of-pocket spending for travel and supportive care, income loss, and long-term disability. Therefore, the inpatient expenditures reported here should be interpreted as a partial estimate of economic burden: they are most useful for hospital budget planning and scenario comparisons, while patient-level financial toxicity requires complementary data sources (surveys, claims linkages, or household cost studies).

The findings indicate that nearly half of inpatient laryngeal cancer spending is already concentrated in patients aged ≥65 years (≈2.38 of 4.85 million USD in 2023), with urban case mix skewed toward older, more resource-intensive episodes. Clinically, this argues for a dual strategy. First, aggressive primary and secondary prevention—systematic smoking-cessation support in ENT and oncology clinics, early dysphonia evaluation pathways, and integration of radon risk assessment in high-burden counties—can flatten the future discharge curve and avoid up to 3.53 million USD in hospital expenditure by 2035 in the Romanian-tariff scenario, with much larger savings if tariffs converge toward EU levels. Second, because aging patients will remain the main cost driver even under optimistic prevention, hospitals must adapt care models: prioritizing geriatric-sensitive anesthesia and rehabilitation, early nutritional and speech-therapy interventions, and multidisciplinary prehabilitation to shorten length of stay and reduce costly complications. In a constrained budget environment, targeting high-risk, older patients for optimized perioperative pathways may offer the best “value for money,” while at the system level, shifting resources from inpatient treatment to prevention is economically rational rather than merely aspirational.

More broadly, recent evaluations of rare-cancer policy implementation and orphan drug access in Southeastern Europe highlight recurring system constraints, including incomplete registries, delayed access pathways, and budget pressure in resource-constrained settings, which are relevant when interpreting administrative-data-based cancer burden studies in the region. Recent analyses of rare cancer policies across Europe and orphan drug access in Bulgaria document similar challenges in Southeastern European health systems, including delayed treatment access, incomplete disease registries, and rising pharmaceutical expenditures in resource-constrained environments [31,32]. Nevertheless, these findings should be interpreted in light of potential residual confounding from unmeasured or incompletely controlled factors, including underlying comorbidities and other patient- and treatment-related characteristics [32].

### 4.2. Study Limitations

This analysis is based on aggregated administrative discharge data and therefore cannot capture individual-level clinical detail such as tumor stage, treatment modality, comorbidity burden, or readmissions, which may substantially influence true cost per case. The cost model uses a top-down approach with assumed Romanian unit tariffs and applies EU and US benchmarks derived from the head-and-neck oncology literature rather than local micro-costing, so absolute values should be interpreted as plausible ranges rather than precise prices. Only direct inpatient medical costs were considered; outpatient visits, radiotherapy delivered in ambulatory settings, rehabilitation, indirect costs (productivity loss, early retirement), and patient out-of-pocket payments were excluded, leading to an underestimation of the full economic burden. High prevalence and temporal variation of “unspecified” subsite coding (C32.9) may introduce misclassification and reduce the interpretability of subsite-specific estimates. Interpretations are limited by the ecological design and the short, pandemic-affected time series; therefore, associations should be viewed as hypotheses for future individual-level linkage studies rather than causal effects. The weak and non-significant discharge–mortality correlation over five annual time points (pandemic-affected) indicates that discharge volume is an imperfect proxy for underlying burden, reinforcing that 2035 outputs should be interpreted as scenario-based budget illustrations rather than epidemiologic forecasts. Scenario projections do not incorporate latency distributions for smoking-related risk reduction, potential future shifts from inpatient to ambulatory care, or integration with GBD/GLOBOCAN incidence models; these limitations may bias the timing and magnitude of projected changes and therefore reinforce interpretation as illustrative budget scenarios. Finally, the short mortality time series (2019–2023) limits inference on the relationship between hospital utilization, survival, and long-term economic outcomes.

The costing approach is another key limitation. The base-case cost was assigned uniformly at the episode level, with only simple age and residence adjustments, because detailed DRG weights, procedures, intensive-care use, length of stay, readmissions, and outpatient treatment costs were unavailable. For this reason, the results should be interpreted as conservative provider-side inpatient budget estimates rather than micro-costed economic evaluations or full societal cost estimates.

The projection component should also be interpreted cautiously. The 2035 values are deterministic scenarios derived from recent utilization and specified prevention multipliers, not probabilistic forecasts. The short 2019–2023 baseline period includes pandemic-related disruption, and longer pre-pandemic and post-pandemic time series would be needed to estimate stable secular growth in laryngeal cancer hospitalizations.

Finally, the high proportion of C32.9 unspecified laryngeal cancer coding limits subsite interpretation. This pattern may reflect documentation and coding practices rather than true tumor localization, and it may obscure clinically important differences between glottic, supraglottic, subglottic, and transglottic disease. We therefore frame subsite results as descriptive and emphasize the need for improved registry linkage and more complete clinical coding.

## 5. Conclusions

In Romania, laryngeal cancer generated approximately 4.85 million USD in provider-side inpatient expenditure in 2023 under current tariff assumptions, while simple re-pricing of the same activity yielded counterfactual totals of 17.20 million USD at an EU benchmark and 132.27 million USD at a US benchmark. These results should be interpreted at the aggregate discharge level. They show that aging, residence-related access patterns, and sustained hospital activity can substantially increase future inpatient budget pressure, whereas smoking reduction, radon mitigation, and systematic evaluation of persistent dysphonia may attenuate—but not eliminate—the projected cost increase. Improved coding specificity, registry linkage, and patient-level costing are needed to refine future estimates and to distinguish utilization, incidence, stage, treatment intensity, and readmission effects.

## Figures and Tables

**Table 1:** One-way sensitivity analysis of projected 2035 inpatient costs under alternative Romanian unit-cost assumptions (USD).

Assumed Mean Cost per Discharge (USD)	2035 Baseline Scenario (11,454 Discharges) Total Cost	2035 with Smoking Reduction (9392 Discharges) Total Cost	2035 Combined Package (8247 Discharges) Total Cost
550 (−50%)	6,299,700	5,165,600	4,535,850
880 (−20%)	10,079,520	8,264,960	7,257,360
1100 (base case)	12,599,400	10,331,200	9,071,700
1320 (+20%)	15,119,280	12,397,440	10,886,040
1650 (+50%)	18,899,100	15,496,800	13,607,550

**Table 2:** Annual laryngeal cancer discharges by sex, 2019–2023.

Year	Total Discharges	Female n (%)	Male n (%)
2019	5408	358 (6.6)	5050 (93.4)
2020	3151	190 (6.0)	2961 (94.0)
2021	3212	156 (4.9)	3056 (95.1)
2022	3876	222 (5.7)	3654 (94.3)
2023	4409	237 (5.4)	4172 (94.6)

**Table 3:** Annual laryngeal cancer discharges by residence, 2019–2023.

Year	Total	Rural n (%)	Urban n (%)
2019	5408	2735 (50.6)	2673 (49.4)
2020	3151	1650 (52.4)	1501 (47.6)
2021	3212	1672 (52.1)	1540 (47.9)
2022	3876	2085 (53.8)	1791 (46.2)
2023	4409	2305 (52.3)	2104 (47.7)

**Table 4:** Age-category distribution of laryngeal cancer discharges by residence, 2019–2023.

Residence	<55 Years n (%)	55–64 Years n (%)	≥65 Years n (%)	Total
Rural	2289 (21.9)	4006 (38.3)	4152 (39.7)	10,447
Urban	1401 (14.6)	3467 (36.1)	4741 (49.3)	9609

**Table 5:** Annual distribution of laryngeal cancer discharges by tumor subsite, 2019–2023.

Year	Glottic	Subglottic	Supraglottic	Transglottic/Other	Unspecified	Total
2019	707	43	217	1862	2579	5408
2020	390	43	106	1205	1407	3151
2021	445	44	110	1072	1541	3212
2022	542	43	166	1066	2059	3876
2023	479	48	181	1440	2261	4409

(Reference categories: 2019, female sex, rural residence, glottic tumors).

**Table 6:** Predictors of age ≥65 years at discharge: grouped binomial logistic regression.

Predictor	Category/Effect	OR	95% CI	*p*-Value
Sex	Male vs. female	1.06	0.94–1.19	0.354
Residence	Urban vs. rural	1.48	1.40–1.57	<0.001
Subsite	Subglottic vs. glottic	0.87	0.66–1.14	0.313
	Supraglottic vs. glottic	0.59	0.50–0.70	<0.001
	Transglottic/other vs. glottic	0.79	0.72–0.87	<0.001
	Unspecified vs. glottic	0.9	0.83–0.98	0.021
Calendar year	Per 1-year increase	1.07	1.06–1.09	<0.001

Abbreviations: OR, odds ratio; CI, confidence interval. Calendar-year effects are estimated over a short 2019–2023 window that includes pandemic-related disruption in admissions and care pathways. The calendar-year OR should therefore be interpreted as a within-period composition trend rather than a long-run secular aging parameter; longer pre-2019 series would be preferable for stable inference.

**Table 7:** Annual laryngeal cancer discharges and age-standardized mortality rates, Romania, 2019–2023.

Year	Discharges (n)	Age-Standardized Mortality (per 100,000)
2019	5408	4.19
2020	3151	4.21
2021	3212	3.76
2022	3876	3.7
2023	4409	3.98

**Table 8:** Estimated national inpatient cost of laryngeal cancer in Romania, 2019–2023, with EU and USA benchmarks (USD).

Year	Discharges	Population	Mean Cost per Discharge—Romania (USD)	Total Inpatient Cost—Romania (USD)	Cost per 100,000 Population—Romania (USD)	Counterfactual Total Cost at EU Rate (USD)	Counterfactual Total Cost at US Rate (USD)
2019	5408	22,205,476	1100	5,948,800	26,800	21,091,200	162,240,000
2020	3151	22,192,107	1100	3,466,100	15,600	12,288,900	94,530,000
2021	3212	22,075,123	1100	3,533,200	16,000	12,526,800	96,360,000
2022	3876	21,970,428	1100	4,263,600	19,400	15,116,400	116,280,000
2023	4409	21,872,785	1100	4,849,900	22,200	17,195,100	132,270,000

Abbreviations: EU, European Union; US, United States; USD, United States dollars. Cost per 100,000 population is reported as a crude budget metric and is not age-standardized.

**Table 9:** Age-stratified inpatient cost of laryngeal cancer in Romania, 2023, with EU/US cost benchmarks (USD).

Age Group (2023)	Discharges	Mean Cost per Discharge—Romania (USD)	Total Inpatient Cost—Romania (USD)	Counterfactual Total Cost at EU Rate (USD)	Counterfactual Total Cost at US Rate (USD)
<55 years	733	1000	733,000	2,858,700	21,990,000
55–64 years	1579	1100	1,736,900	6,158,100	47,370,000
≥65 years	2097	1135	2,380,100	8,178,300	62,910,000

Abbreviations: EU, European Union; US, United States; USD, United States dollars.

**Table 10:** Rural–urban differences in inpatient laryngeal cancer costs, Romania 2023 (USD).

Residence	Discharges 2023	Mean Cost per Discharge—Romania (USD)	Total Inpatient Cost—Romania (USD)	Counterfactual Total Cost at EU Rate (USD)	Counterfactual Total Cost at US Rate (USD)
Rural	2305	1050.00	2,420,200	8,989,500	69,150,000
Urban	2104	1154.78	2,429,600	8,205,600	63,120,000
Total	4409	—	4,849,900	17,195,100	132,270,000

Abbreviations: EU, European Union; US, United States; USD, United States dollars; —, not applicable.

**Table 11:** Projected inpatient cost of laryngeal cancer hospitalizations in Romania, 2035, under alternative scenarios (USD).

Scenario	Projected Discharges in 2035	Mean Cost per Discharge—Romania (USD)	Total Inpatient Cost—Romania (USD)	Counterfactual Total Cost at EU Rate (USD)	Counterfactual Total Cost at US Rate (USD)	Incremental Cost vs. 2023—Romania (USD)
Observed 2023 baseline	4409	1100	4,849,900	17,195,100	132,270,000	0
2035 baseline scenario (no new policies)	11,454	1100	12,599,400	44,670,600	343,620,000	7,749,500
2035 with 30% smoking reduction	9392	1100	10,331,200	36,628,800	281,760,000	5,481,300
2035 with smoking + radon mitigation + dysphonia screening	8247	1100	9,071,700	32,163,300	247,410,000	4,221,800

Abbreviations: EU, European Union; US, United States; USD, United States dollars.

**Table 12:** Discharge-to-patient ratio sensitivity: implied unique hospitalized patients and per-patient inpatient cost.

Scenario	Discharges	Assumed Mean Discharges per Patient	Implied Unique Hospitalized Patients (≈Discharges/Ratio)	Implied Inpatient Cost per Patient (≈1100 × Ratio), USD
Observed 2023 baseline	4409	1	4409	1100
		1.1	4008	1210
		1.2	3674	1320
		1.3	3392	1430
2035 baseline scenario	11,454	1	11,454	1100
		1.1	10,413	1210
		1.2	9545	1320
		1.3	8811	1430
2035 with smoking reduction	9392	1	9392	1100
		1.1	8538	1210
		1.2	7827	1320
		1.3	7225	1430
2035 combined package	8247	1	8247	1100
		1.1	7497	1210
		1.2	6873	1320
		1.3	6344	1430

**Table 13:** Precision sensitivity for the grouped binomial logistic regression: confidence intervals after inflating standard errors to reflect plausible within-hospital/-county clustering.

Predictor (Reference)	OR (Primary Model)	95% CI (Primary)	95% CI with SE × 1.2	95% CI with SE × 1.4
Urban vs. rural	1.48	1.40–1.57	1.38–1.59	1.37–1.60
Calendar year (per +1 year)	1.07	1.06–1.09	1.05–1.09	1.05–1.09
Supraglottic vs. glottic	0.59	0.50–0.70	0.48–0.72	0.47–0.75
Transglottic/other vs. glottic	0.79	0.72–0.87	0.71–0.89	0.69–0.90
Unspecified vs. glottic	0.9	0.83–0.98	0.82–0.99	0.80–1.01

**Table 14:** Range-based benchmark re-pricing of 2023 activity using alternative EU and US unit-cost ranges.

Benchmark Setting	Assumed Unit Cost per Discharge (USD)	Counterfactual 2023 Total Cost (USD)
EU (lower bound)	3000	13,227,000
EU (base case used in manuscript)	3900	17,195,100
EU (upper bound)	5000	22,045,000
US (lower bound)	20,000	88,180,000
US (base case used in manuscript)	30,000	132,270,000
US (upper bound)	40,000	176,360,000

## Data Availability

The data presented in this study are available on request from the corresponding author. The data are not publicly available due to ethical restrictions and institutional policies.

## Appendix A

**Table A1:** Year-by-year projected discharge volumes and scenario multipliers, 2024–2035.

Year	Baseline Discharges	Smoking Multiplier	Smoking Scenario	Combined Multiplier	Combined Scenario
2024	4774	0.985	4702	0.977	4663
2025	5169	0.970	5014	0.953	4928
2026	5598	0.955	5346	0.930	5206
2027	6061	0.940	5697	0.907	5495
2028	6563	0.925	6071	0.883	5797
2029	7106	0.910	6467	0.860	6111
2030	7695	0.895	6887	0.837	6438
2031	8332	0.880	7332	0.813	6777
2032	9022	0.865	7804	0.790	7127
2033	9769	0.850	8304	0.767	7490
2034	10,578	0.835	8833	0.743	7863
2035	11,454	0.820	9392	0.720	8247

Table A1 operationalizes the deterministic projection model requested by the reviewer and reports the exact multipliers used to obtain the published 2035 scenario totals.

**Table A2:** Sensitivity analyses for high unspecified-subsite coding (C32.9).

Model Specification	Urban vs. Rural (Age ≥ 65)	Calendar Year Effect	Interpretation
Primary model (sex + residence + subsite + year)	OR 1.48 (95% CI 1.40–1.57)	OR 1.07 per year (95% CI 1.06–1.09)	Main analysis reported in Table 6
Excluding C32.9 strata	Positive; remained statistically significant	Positive; remained statistically significant	Core inference unchanged
Excluding tumor subsite as a covariate	Positive; remained statistically significant	Positive; remained statistically significant	Core inference unchanged
Restricting to specified subsites only	Positive; remained statistically significant	Positive; remained statistically significant	Core inference unchanged

Across all alternative specifications, the coefficients retained the same direction and significance pattern, supporting the robustness of the main ecological inference (Table A2).
